# Coverage‐ and Temperature‐Dependent Rates of Metalation, Ring Fusion, and Polymerization of Benzoporphyrins on Cu(111)

**DOI:** 10.1002/chem.202500998

**Published:** 2025-07-01

**Authors:** Maximilian Muth, Majid Shaker, Julien Steffen, Alexander Wolfram, Simon Steinbach, Lampros‐Pascal Gazetas, Norbert Jux, Andreas Görling, Hans‐Peter Steinrück, Ole Lytken

**Affiliations:** ^1^ Lehrstuhl für Physikalische Chemie II Friedrich‐Alexander‐Universität Erlangen‐Nürnberg Egerlandstr. 3 91058 Erlangen Germany; ^2^ Lehrstuhl für Theoretische Chemie Friedrich‐Alexander‐Universität Erlangen‐Nürnberg Egerlandstr. 3 91058 Erlangen Germany; ^3^ Lehrstuhl für Organische Chemie II Friedrich‐Alexander‐Universität Erlangen‐Nürnberg Nikolaus‐Fiebiger‐Str. 10 91058 Erlangen Germany; ^4^ Erlangen National High Performance Computing Center (NHR@FAU) Martensstr. 1 91058 Erlangen Germany

**Keywords:** Cu(111), metalation, porphyrins, STM, TPD

## Abstract

Using scanning‐tunneling microscopy (STM), temperature‐programmed desorption (TPD), X‐ray photoelectron spectroscopy (XPS), and density‐functional theory (DFT), we have investigated metalation, ring fusion, and polymerization of three different benzoporphyrins, tetraphenyltransdisbenzoporphyrin (2‐Benzo), copper‐tetraphenyltransdibenzoporphyrin (Cu 2‐Benzo), and tetraphenyltetrabenzoporphyrin (4‐Benzo), from 280 to 1000 K on Cu(111), and compare with previous data of tetraphenylporphyrin (0‐Benzo) on Cu(111). Using STM, we observe 2‐Benzo to adsorb as individual, rather‐immobile molecules at room temperature, which coalesce into elongated islands of metalated molecules at 373 K. N 1 s XPS spectra confirm the metalation, and DFT‐calculated structures and simulated STM images confirm the appearance in STM. Upon further heating to 398 K, the islands become rough and irregularly shaped, before completely breaking up at 423 K, most likely related to intramolecular ring fusion. Heating to 950 K leads to complete polymerization and a rough structure on the surface. Using H_2_ TPD, we follow the coverage‐dependent rates of metalation, ring fusion, and polymerization for all four molecules. The metalation rate is found to correlate with, sometimes abrupt, changes in the adsorption structures, whereas the rates of ring fusion and polymerization decrease gradually with increasing coverage, for all molecules.

## Graphical abstract

Using temperature‐programmed desorption, supported by scanning tunneling microscopy, X‐ray photoelectron spectroscopy, and density‐functional theory, we have investigated metalation, ring fusion, and polymerization of three different benzoporphyrins on Cu(111). We furthermore compare our results with previous data of tetraphenylporphyrin on Cu(111).

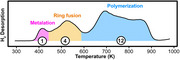

## 1 Introduction

Porphyrins are strongly‐colored semiconducting organic molecules^[^ ^1^, ^2^ ^]^ with interesting electronic,^[^ ^3^, ^4^, ^5^, ^6^, ^7^, ^8^, ^9^ ^]^ catalytic,^[^ ^10^, ^11^, ^12^, ^13^, ^14^, ^15^, ^16^, ^17^ ^]^ and sensing properties.^[^ ^18^, ^19^, ^20^, ^21^ ^]^ On solid surfaces, they are known to undergo multiple reactions as a function of temperature, such as metalation,^[^ ^22^, ^23^, ^24^, ^25^, ^26^, ^27^, ^28^, ^29^, ^30^, ^31^, ^32^, ^33^, ^34^ ^]^ transmetalation,^[^ ^35^, ^36^, ^37^, ^38^, ^39^, ^40^ ^]^ ring fusion,^[^ ^41^, ^42^, ^43^, ^44^ ^]^ and polymerization.^[^ ^43^, ^44^ ^]^

Metalation occurs when a free‐base porphyrin molecule reacts with either codeposited metal atoms,^[^ ^45^, ^46^ ^]^ with metal atoms from a metal substrate,^[^ ^22^, ^23^, ^24^, ^25^, ^26^, ^27^, ^28^, ^29^, ^30^ ^]^ or with metal ions from a metal‐oxide substrate,^[^ ^31^, ^32^, ^33^, ^34^ ^]^ producing a metalloporphyrin. The reaction can be spontaneous near room temperature,^[^ ^30^, ^33^, ^34^ ^]^ or require modest heating.^[^ ^22^, ^23^, ^24^, ^25^, ^26^, ^27^, ^28^, ^29^, ^31^, ^32^ ^]^ Metalation can be seen as belonging to a larger group of coordination reactions, where ligands coordinate surface atoms. There are many very well‐known wet‐chemical examples, where ligand‐metal coordination plays a major role, such as cyano groups coordinating to cobalt adatoms, which can be used to build covalent organic frameworks^[^ ^47^ ^]^ or 2D‐networks.^[^ ^48^ ^]^ Similar ligand‐metal interactions are also used for metal‐coordinated intermediate steps in catalysis. One prominent example is the onsurface Ullmann coupling reaction,^[^ ^49^ ^]^ where aryl halides first coordinate to a copper atom before a carbon–carbon bond is formed. In a similar way, boronic acids, and organo halides coordinate to palladium in Suzuki coupling,^[^ ^50^ ^]^ which is used to cross‐couple two organic fragments. An additional example, involving the coordination of a palladium atom in a catalyzed reaction, is the Sonogashira coupling.^[^ ^51^ ^]^

Transmetalation, where one metal center is exchanged for another, is a well‐known reaction for dissolved porphyrin molecules in solution, but has also been observed for Zn‐tetraphenylporphyrin adsorbed on Au(111) under ultrahigh vacuum and subsequently exposed to Cu^2+^ ions in solution.^[^ ^35^ ^]^ It has been observed in ultrahigh vacuum for Pb‐tetraphenylporphyrin on Cu(111) with temperature‐programmed desorption (TPD), as desorption of Cu‐ tetraphenylporphyrin from the surface upon annealing,^[^ ^36^ ^]^ but has also been suggested for other systems, based on stark shifts in the binding‐energy positions of the metal centers in X‐ray photoelectron spectroscopy (XPS).^[^ ^37^, ^38^, ^39^, ^40^ ^]^

Ring fusion is a dehydrogenation reaction, forming additional carbon–carbon bonds within the molecules. In solution chemistry, the ring fusion of porphyrins can also be catalyzed by palladium.^[^ ^52^, ^53^, ^54^, ^55^ ^]^ Another approach is the dehydrogenation via the Scholl oxidation.^[^ ^56^, ^57^ ^]^ On surfaces, ring fusion typically occurs at elevated temperatures on both metal^[^ ^41^, ^42^, ^43^, ^44^, ^58^ ^]^ and oxide^[^ ^59^, ^60^ ^]^ surfaces. It was first observed with NEXAFS on Ag(111),^[^ ^61^ ^]^ but in the meantime very‐high quality scanning‐tunneling microscopy (STM) images exist, showing the variety of ring‐fused species formed on the surfaces.^[^ ^23^, ^36^, ^44^, ^62^ ^]^

**Figure 1:**
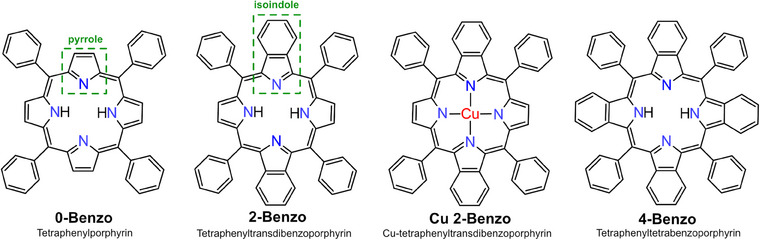
The four molecules used in this study. The green dashed boxes indicate the pyrrole and isoindole structural elements, referred to throughout the text.

In this study, we have used STM, density‐functional theory (DFT) calculations, XPS, and TPD to follow the coverage‐dependent thermal reactions (metalation, ring fusion, and polymerization) from 280 to 1000 K of three benzoporphyrins with Cu(111): tetraphenyltrandisbenzoporphyrin (2‐Benzo), copper‐tetraphenyltransdibenzoporphyrin (Cu 2‐Benzo), and tetraphenyltetrabenzoporphyrin (4‐Benzo). Furthermore, we compare their reactions to previous data of tetraphenylporphyrin (0‐Benzo) on Cu(111) published by Röckert et al.^[^ ^22^, ^63^ ^]^ The structures of all discussed molecules are shown in Figure 1.

In comparison to normal tetraphenylporphyrins, benzoporphyrins possess additional benzo groups fused to their pyrrole moieties. The fused pyrrole and benzo groups together form an isoindole moiety (see dashed boxes in Figure 1). Because of their enlarged conjugated system, benzoporphyrins have received interest in the field of electronics and electro‐optics.^[^ ^64^, ^65^, ^66^ ^]^ Extending the π‐system of the porphyrin macrocycle allows us to further tailor the properties, for example for light absorption at specific wave lengths, which is relevant for more efficient solar cells,^[^ ^67^, ^68^, ^69^ ^]^ or for tuning near‐infrared phosphorescence for improved imaging techniques in medicine.^[^ ^70^ ^]^

## 2 Experimental Section

The measurements were performed in two separate ultrahigh‐vacuum (UHV) setups. STM was done at room temperature in a UHV chamber with a base pressure in the low 10^−10^ mbar range. The chamber is equipped with a variable‐temperature microscope (RHK UHV VT STM 300 and RHK SPM 1000 electronics). A Pt/Ir tip in constant current mode was used with the bias voltage applied to the sample. The STM images were processed in Gwyddion.^[^ ^71^ ^]^

XPS was performed in a separate UHV chamber with a base pressure in the mid 10^−10^ mbar range, a monochromatized Al K_α_ X‐ray source (Scienta, SAX 100, hν = 1486.6 eV) and a hemispherical electron analyzer (Scienta, SES 200).

H_2_ TPD was performed in the preparation chamber of the XPS setup with a Balzers QMA 400 quadrupole mass spectrometer, mounted inside a copper Feulner cup,^[^ ^72^ ^]^ with the ø5 mm opening of the cup placed 0.5 mm above the surface of the ø10 mm sample. The distance is controlled by bringing the edge of the sample holder into electrical contact with the Feulner cup, retracting the sample holder by 1 mm, centering the sample directly under the opening of the Feulner cup, and then reapproaching the sample holder by 1 mm. Because the sample holder is a very rigid design with the surface of the sample always exactly 0.5 mm below the surface of the sample holder, this defines the distance to the Feulner cup. The narrow gap between the sample and the Feulner cup results in a low conductance, which strongly increases the partial pressure in the mass spectrometer of molecules desorbing from the front of the sample, relative to molecules from the sample holder, and sides and back of the sample. In addition, a relatively‐fast heating rate of 5 K/s was used to keep the partial pressure of the desorbing molecules significant, relative to the background of H_2_ in the mass spectrometer.

Because H_2_ is a very light molecule, with a high flux through any given cross section, it has a very low residence time and therefore low partial pressure in the mass spectrometer. To increase the partial pressure in the mass spectrometer, we let the narrow gap between the sample and the Feulner cup act as the only differential pumping of the mass spectrometer. This means it is critical that we reproduce the distance between the Feulner cup and the sample every time to keep the partial pressures inside the mass spectrometer reproducible. For larger molecules, additional differential pumping would be needed to prevent too‐long residence times.

For the TPD measurements, the Cu(111) single‐crystal was mounted with two tantalum heating wires (*ø* = 0.40 mm) used for resistive heating, and the temperature of the sample was measured with a thin type‐K thermocouple (*ø* = 0.125 mm) mounted inside a hole in the side of the crystal. The other ends of the tantalum heating wires were attached directly to two large liquid‐nitrogen‐cooled copper blocks, keeping the temperature increase of the sample holder below 6 K during the TPD measurements, thereby minimizing desorption from the sample holder. In addition, a mask with an opening of *ø* = 8 mm, slightly smaller than the sample (*ø* = 10 mm), was placed in front of the sample during deposition of the porphyrin molecules, ensuring that no porphyrin molecules were deposited on the heating wires or the sample holder.

Before every XPS and TPD experiment, the Cu(111) single crystal was cleaned by 1 kV Ar^+^‐ion sputtering for 30 minutes at room temperature, followed by annealing at 850 K for 30 minutes. For the STM experiments, which were performed in a separate chamber, the sample was cleaned by 600 V Ar^+^‐ion sputtering for 15 minutes, followed by annealing at 850 K for 1 to 5 minutes.

Free‐base tetraphenyltransdibenzoporphyrin (2‐Benzo) and tetrabenzoporphyrin (4‐Benzo)^[^ ^73^ ^]^ as well as Cu 2‐Benzo, see Figure 1, were synthesized by the group of Prof. Norbert Jux at the Universität Erlangen–Nürnberg. Because of the statistical synthesis route and difficult purification, both the 2‐Benzo and Cu 2‐Benzo molecules contain monobenzo impurities (mass spectra of 2‐Benzo and Cu 2‐Benzo can be found in Figure S2 in the Supporting Information).

For the XPS and TPD experiments, the porphyrins were evaporated from separate radiatively‐heated graphite crucibles at temperatures ranging from 490 – 550 K, at a distance of 4 cm from the sample. For the STM measurements, conducted in a separate UHV chamber, the 2‐Benzo molecules were evaporated at a longer distance from the sample (∼20 cm) from quartz crucibles at a temperature of 630 K. After filling the evaporators with fresh molecules, the molecules were degassed, first in ultrahigh vacuum at 373 K for 16 hours, and subsequently at the evaporation temperatures for at least 30 minutes. Based on the amount of degassing we estimate the concentration of monobenzo species in the deposited layers to be roughly 5 – 15%, see Figure S2 in the Supporting Information.

For the STM measurements, evaporation rates were calibrated by counting the free‐base molecules in the STM images at low coverages, where individual rather‐immobile molecules are clearly visible. A video of the mobility of 2‐Benzo molecules on Cu(111) can be found in the Supporting Information of Shaker et al.^[^ ^30^ ^]^ For longer deposition times, resulting in higher coverages, the evaporation rate was assumed to be constant. Molecule counting works well for free‐base molecules, but — due to their higher mobility on the surface — will underestimate the coverage of metalated molecules.

For the XPS and TPD measurements, relative coverages were measured as C 1 s : Cu 2*p*_3/2_ intensity ratios and converted into absolute coverages in molecules/nm^2^ by comparing with C 1 s : Cu 2*p*_3/2_ intensity ratios of known coverages, measured with STM, of free‐base tetraphenylporphyrin on Cu(111) from a previous study,^[^ ^22^ ^]^ taking the different number of carbon atoms in our four molecules into account. In a previous publication^[^ ^30^ ^]^ comparing coverage‐dependent rates of metalation of 2‐Benzo on Cu(111), using the same coverage calibrations, we obtained a good agreement between STM and XPS data.

For details on the DFT‐calculations, we refer to the Supporting Information of our previous publication.^[^ ^30^ ^]^

## 3 Results and Discussion:

### 3.1 Metalation

**Figure 2:**
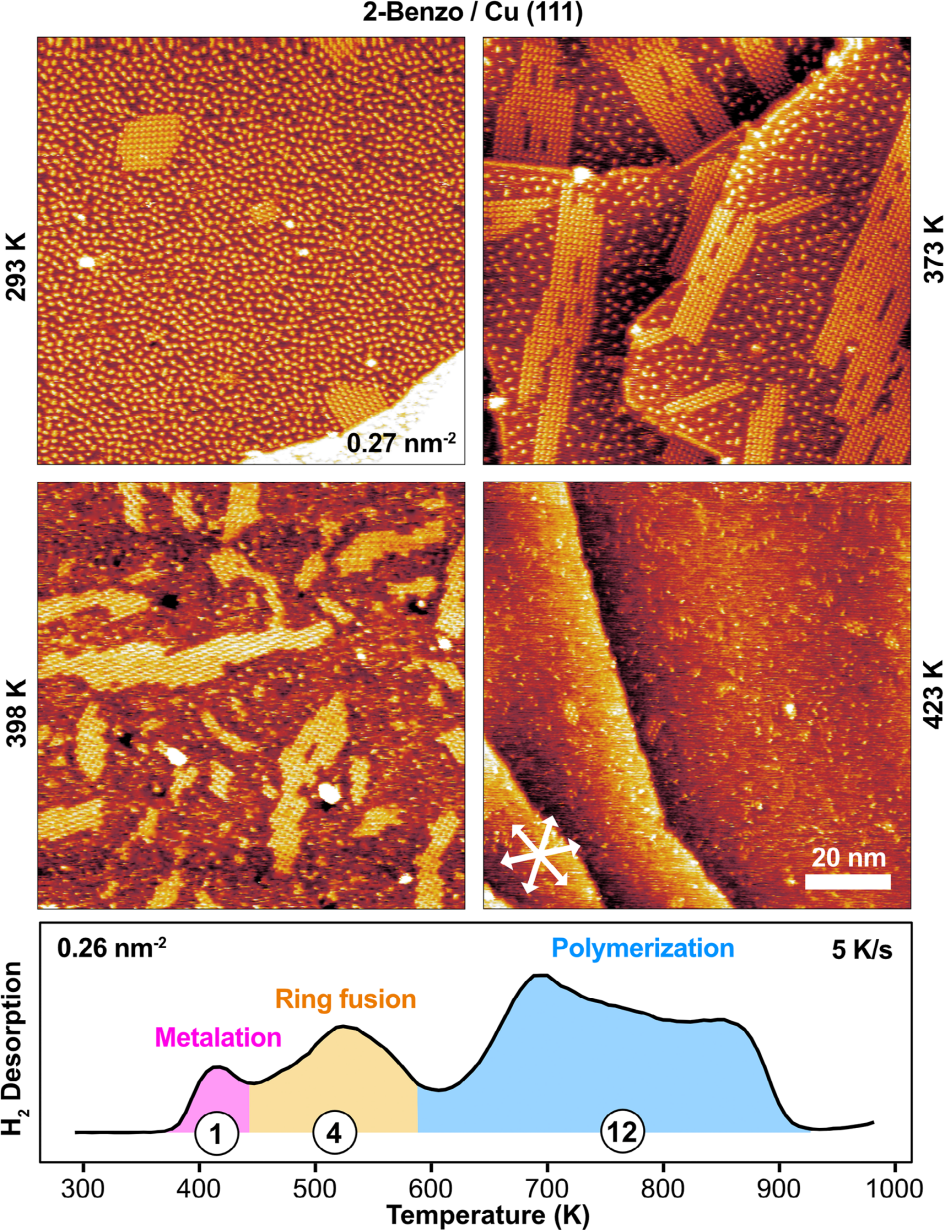
STM images of 0.27 molecules/nm^2^ of 2‐Benzo on Cu(111) after 1 day at room temperature (293 – 300  K), and after annealing to 373 K (1 minute), 398 K (flashed), and 423 K (5 minutes). (293 K: −0.99 V, 62 pA, 373 K: −1.01 V, 32 pA, 398 K: −0.23 V, 42 pA, 423 K: −0.75 V, 39 pA). For the images at 373 and 423 K, a polynomial background was subtracted to better show the islands across all the terraces. We attribute the island formation to metalation and the roughening and breaking up of the islands at higher temperatures to ring fusion. Shown is also a TPD spectrum recorded at the same coverage as the STM images.

Figure 2 shows STM images of 0.27 2‐Benzo molecules/nm^2^ deposited on Cu(111) at room temperature (293 – 300 K) and heated stepwise to 423 K. At room temperature, the molecules are present as individual, rather‐immobile molecules in a disordered arrangement, with a few small ordered islands. As the temperature is increased to 373 K, the ordered islands grow in size and become elongated, while the density of individual immobile molecules decreases and stripy features appear in between the immobile molecules.

In two recent publications from our group,^[^ ^30^, ^74^ ^]^ we have identified the individual, isolated molecules present on the surface as intact, free‐base 2‐Benzo molecules, and the ordered islands as metalated Cu 2‐Benzo molecules, held together by T‐type interactions.

**Figure 3:**
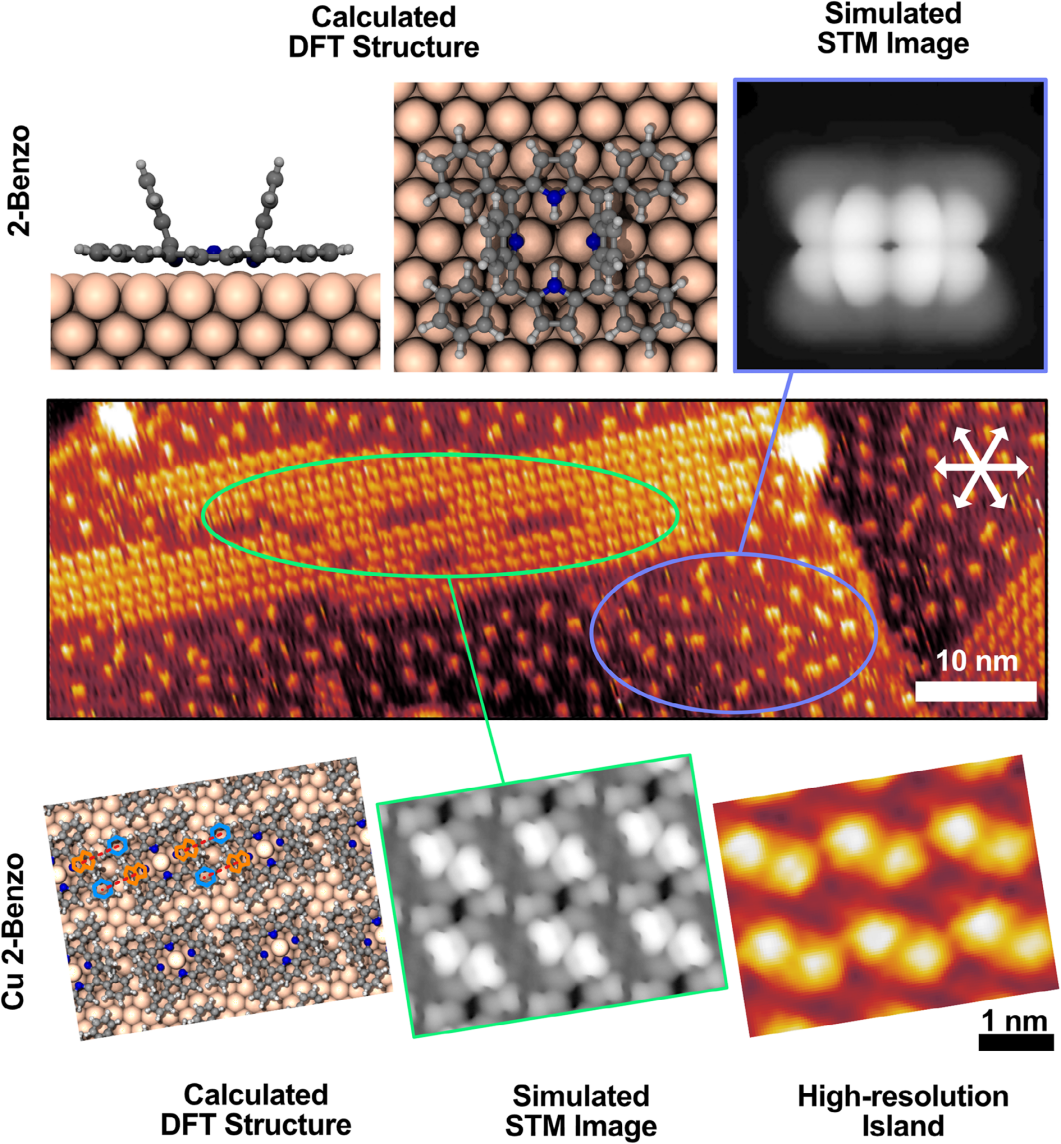
Assignment of individual elongated protrusions and the double‐protrusion island structure to the geometry‐optimized structures and simulated STM images of 2‐Benzo and Cu 2‐Benzo on Cu(111), calculated by DFT in our previous publication.^[^ ^30^ ^]^ The STM image shown in the middle was cropped and rotated from the 373 K measurement of Figure 2 (−1.01 V, 32 pA). In the calculated DFT structure of the Cu 2‐Benzo islands, the T‐type interactions between the isoindole (orange) and phenyl moieties (blue) of one molecule with the neighboring molecules are exemplarily marked. A magnified image of the calculated structure can be found in the Supporting Information Figure S3. (Cu 2‐Benzo high‐resolution island: −1.02 V, 33 pA).

The isolated, free‐base 2‐Benzo molecules adopt a strongly‐distorted inverted structure on the Cu(111) surface, see Figure 3 and Shaker et al.^[^ ^30^ ^]^ In this strongly‐distorted structure, the isoindole groups of the free‐base molecules are orientated nearly perpendicular to the surface, forming strong bonds between the nitrogen atoms of the isoindole groups and copper atoms in the surface. Such conformations have been seen for many other free‐base porphyrins on Cu(111), and result in rather‐immobile molecules, which can be imaged by STM at room temperature.^[^ ^75^, ^76^, ^77^, ^78^, ^79^, ^80^, ^81^ ^]^ Simulated STM images of the strongly, distorted free‐base 2‐Benzo structure, see Figure 3, show that the main contrast is given by the upwards‐pointed isoindole moieties, and the elongated single protrusions we observe experimentally for the isolated molecules agree well with the simulated images.

The Cu 2‐Benzo islands on Cu(111) were found to consist of molecular rows, see Figure 3 and Steffen et al.,^[^ ^74^ ^]^ held together by T‐type interactions between the flat‐lying phenyl rings and the more upright‐standing isoindole groups, see Figure S3. Simulated STM images of this structure predict the upright‐standing isoindole groups to appear as separate protrusions, in very‐good agreement with our experimentally, measured STM images, see Figure 3.

Based on those two publications,^[^ ^30^, ^74^ ^]^ we therefore assign the disordered individual molecules in Figure 2 to free‐base 2‐Benzo molecules and the ordered islands to metalated Cu 2‐Benzo molecules, formed as the free‐base 2‐Benzo molecules react with the copper surface.

The stripy features visible in between the individual molecules in Figure 3, starting at 373 K and becoming more pronounced at 398 and 423 K, are likely highly‐mobile metalated Cu 2‐Benzo molecules in equilibrium with the ordered Cu 2‐Benzo islands, consistent with what has been observed previously for many other adsorbed metalloporphyrins.^[^ ^24^, ^26^, ^82^ ^]^

**Figure 4:**
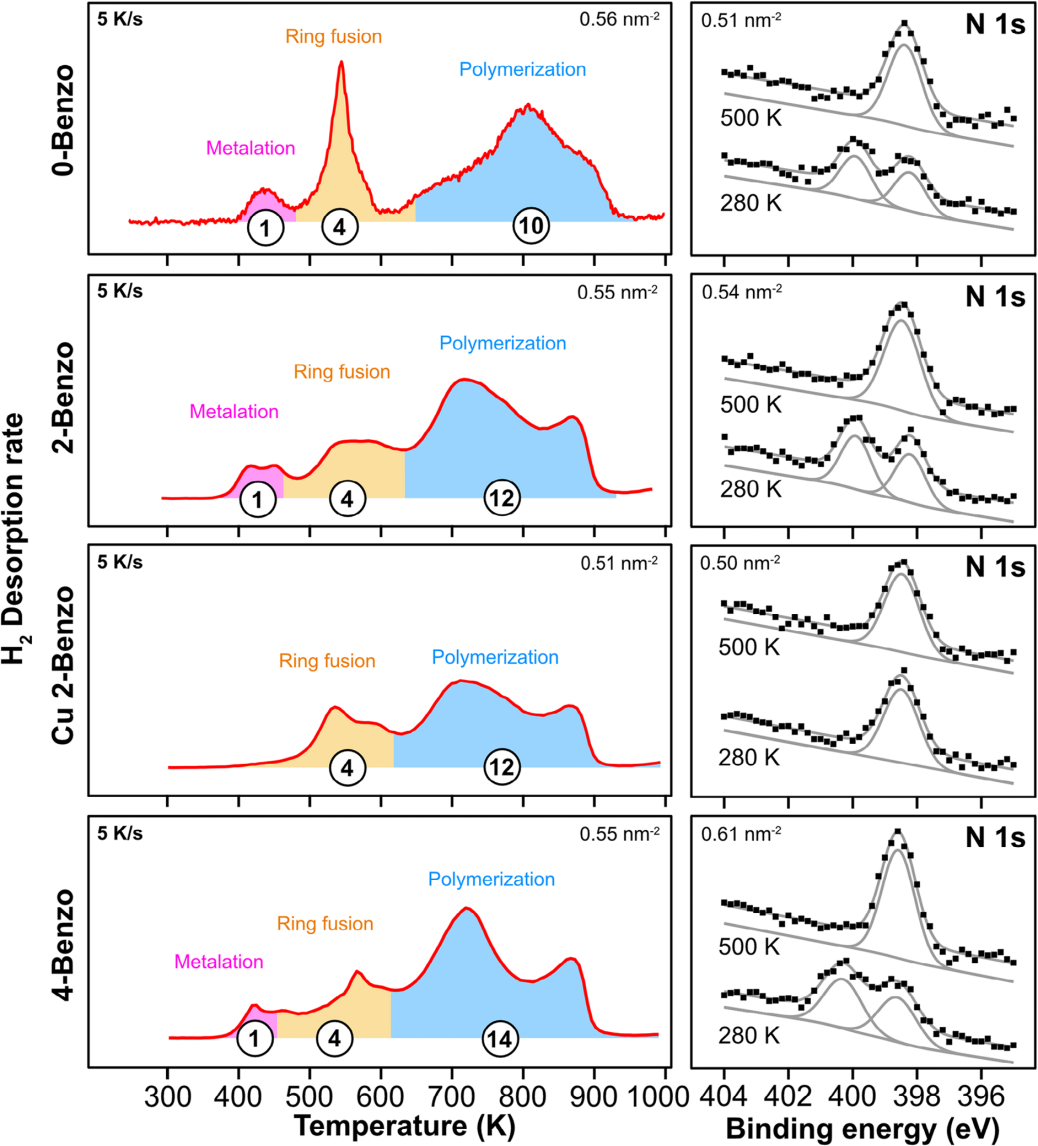
(left) TPD spectra of fully closed layers, the spectrum of 0‐Benzo was previously published by Röckert et al.^[^ ^63^ ^]^ The colored areas under the curves are proportional to the expected H_2_ desorption ratio of metalation, ring fusion, and polymerization (e.g., 1 : 4 : 12 for 2H‐Benzo). (Right) N 1 s XPS region of the respective porphyrins directly after deposition at 280 K and again after heating to 500 K at 5 K/s and immediately cooling back down. For all three free‐base molecules, after heating to 500 K, the N 1 s spectra have changed from the two N 1 s peaks characteristic of free‐base porphyrins, aminic (─NH─) at ∼400.0 eV and iminic (─N =) at ∼398.5 eV, to the single N 1 s peak at ∼398.5 eV characteristic of a metalloporphyrin, where all four nitrogen atoms of the molecule are equivalent.

Metalation with the copper substrate is consistent with our N 1 s XPS spectra of all three free‐base molecules (0‐Benzo, 2‐Benzo, and 4‐Benzo), see Figure 4 (right panel), which after heating to 500 K at 5 K/s and immediately cooling back down, have changed from the two N 1 s peaks characteristic of a free‐base porphyrin (─NH─ at ∼400.0 eV and ─N = at ∼398.5 eV) into the four equivalent nitrogen atoms, and thus single N 1 s peak at ∼398.5 eV, of a metalloporphyrin.

**Figure 5:**
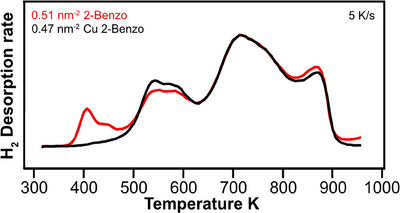
Direct comparison of the H_2_‐TPD spectra of free‐base 2‐Benzo and metalated Cu 2‐Benzo on Cu(111). The additional H_2_ desorption for 2‐Benzo around 400 K, not present for Cu 2‐Benzo, is indicative of metalation, where the two aminic protons in the center of the molecule are replaced by a copper atom from the surface and desorb as H_2_. Both spectra were normalized to have the same maximum intensity for better comparison.

The metalation reaction is also clearly visible in TPD in the desorption rate of H_2_, see Figures 2 and  4 (left panel). As the free‐base molecules metalate, the hydrogen atoms of the aminic (─NH─) nitrogen atoms are transferred to the Cu(111) surface, where they eventually combine into H_2_, which desorbs.^[^ ^83^ ^]^ When directly comparing the desorption rates of H_2_ for 2‐Benzo and Cu 2‐Benzo from 280 to 1000 K, see Figure 5, it is clear that the two desorption curves are almost identical, except for the initial desorption between 375 and 475 K for 2‐Benzo. This is consistent with metalation of 2‐Benzo with copper atoms from the Cu(111) surface, creating Cu 2‐Benzo and releasing one hydrogen molecule per adsorbed 2‐Benzo molecule.

As mentioned in the introduction, metalation is a very common reaction for free‐base porphyrin molecules adsorbed on surfaces and has been observed for codeposited metal atoms, such as cobalt^[^ ^46^ ^]^ or iron,^[^ ^45^ ^]^ for metal atoms from metal substrates, such as Cu(111),^[^ ^22^, ^23^, ^24^, ^25^, ^26^, ^27^, ^28^, ^29^, ^30^ ^]^ Fe(110), and Ni(111),^[^ ^84^ ^]^ for metal ions from oxide substrates, such as MgO,^[^ ^33^, ^85^ ^]^ rutile TiO_2_,^[^ ^31^, ^32^ ^]^ and cobalt oxides,^[^ ^34^ ^]^ and even for metal ions from solutions.^[^ ^86^ ^]^

### 3.2 Ring Fusion

As the surface is heated further to 398 K, STM shows that the ordered islands become rough and irregular and begin to break apart into smaller islands, see Figure 2. The amount of individual immobile molecules decreases even further, and the amount of stripy features from highly mobile molecules increases significantly. Upon further heating to 423 K, the islands have almost completely broken up and stripy features dominate the STM image.

These changes are most likely caused by a ring‐fusion reaction, creating additional carbon–carbon bonds within the molecules, a behavior that is very similar to the fusion on Au(111), Ag(111), and Cu(111) of anthracene polymer chains, created from Ullmann coupling of DBBA (10,10′‐dibromo‐9,9′‐bianthryl) and related molecules, into graphene nanoribbons.^[^ ^87^ ^]^

For adsorbed porphyrin molecules, the ring‐fusion reaction was first observed with NEXAFS on Ag(111)^[^ ^61^ ^]^ and STM on Cu(111).^[^ ^23^ ^]^ In the meantime, it has also been reported for rutile TiO_2_(110),^[^ ^59^, ^60^ ^]^ and very high‐quality STM images are now available for zinc‐tetraphenylporphyrin on Ag(111) and Ag(100),^[^ ^44^, ^58^ ^]^ fluorinated tetraphenylporphyrins on Au(111),^[^ ^88^ ^]^ and cobalt‐meso‐tetra(p‐methoxyphenyl)porphyrin on Au(111),^[^ ^89^ ^]^ showing the full range of isomers formed. These ring‐fused species are often highly, mobile and require low temperatures^[^ ^58^, ^88^, ^89^ ^]^ or crosslinking at high coverages^[^ ^23^, ^44^, ^58^ ^]^ to be imaged.

Benzoporphyrins and ring‐fused porphyrins both belong to a group of porphyrins with extended π‐systems, which increase basicity, decrease their oxidation potentials, but also red‐shift the optical spectra, improving their efficiency in solar cells.^[^ ^55^, ^90^, ^91^, ^92^ ^]^

**Figure 6:**
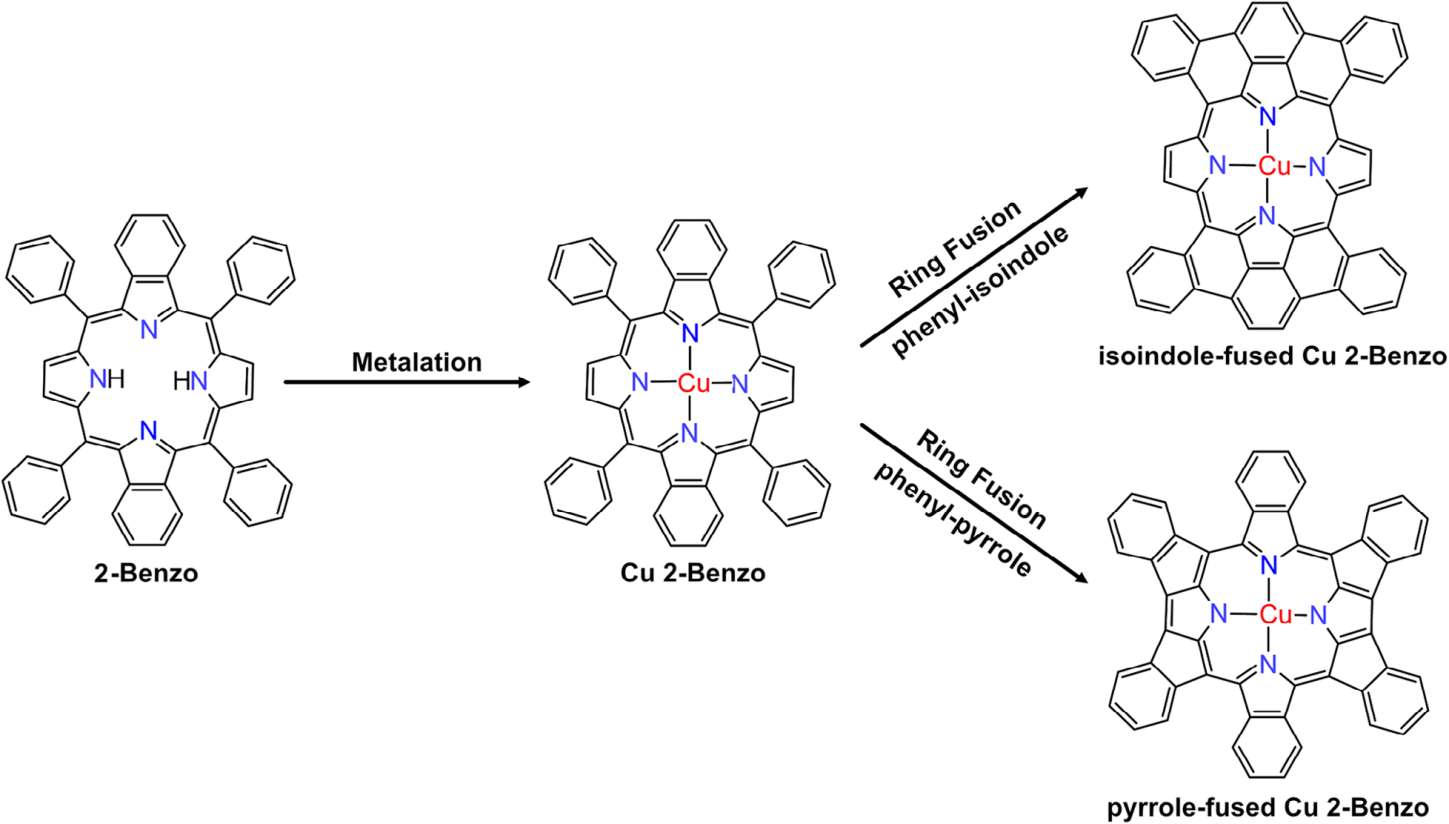
The metalation of 2‐Benzo to Cu 2‐Benzo, followed by ring fusion. Ring fusion can occur between phenyl and isoindole groups or phenyl and pyrrole groups, and various isomers can be formed. However, because of the high mobility of the ring‐fused species on the Cu(111) surface, we are not able to identify the isomers formed by STM.

For Cu 2‐Benzo, ring fusion could occur between the phenyl rings and the isoindole groups or the phenyl rings and the pyrrole groups, see Figure 6. Unfortunately, because of the high mobility of the ring‐fused species on the Cu(111) surface, we are not able to identify the isomers formed on the surface with our room‐temperature STM setup. A ring fusion between the phenyl rings and the isoindole groups would flatten both groups and make the T‐type interaction between adjacent molecules impossible, but even a ring fusion between the phenyl rings and the pyrrole groups would flatten the phenyl rings and make the interaction less favorable. This is likely the cause of the eventual breakup of the ordered islands.

The T‐type interactions within the rows, see Figure 3, mean it costs energy to terminate the molecular rows, causing the elongated shape of the Cu 2‐Benzo islands. However, in the presence of partially‐ring‐fused molecules, which can only have T‐type interactions in one direction, the rows will likely be terminated by these partially‐ring‐fused molecules, lowering the energy cost of terminating the molecular rows. This would cause the islands to become rougher in shape, in agreement with what we experimentally observe at 398 K, see Figure 2. As the reaction proceeds and most molecules have undergone at least one ring fusion, only dimer formation is possible and the islands fall apart, leaving only highly, mobile molecules on the surface.

Any ring‐fusion reaction will result in desorption of H_2_ and will therefore be clearly visible in TPD. If we look at the desorption of H_2_ from 0.26 molecules/nm^2^ of 2‐Benzo in Figure 2, we can identify three regions, roughly from 400 – 450 K (shaded in magenta), 450 – 600 K (orange), and 600–900 K (blue). We know the first reaction is metalation, resulting in the desorption of one H_2_ molecule per porphyrin molecule. The second reaction is likely ring fusion, and because more than four ring fusions would curve the molecule and eventually result in a bowl‐shaped molecule (with significant strain), we only expect desorption of four H_2_ molecules per porphyrin molecule for the ring fusion.

### 3.3 Polymerization

As the temperature is increased further, higher and higher activation energies can be overcome and more and more carbon‐hydrogen bonds will begin to break, and as soon as atomic hydrogen is formed on the surface, it will combine and desorb as H_2_. The reaction is therefore irreversible, and as soon as the temperature is sufficient to overcome the activation energy of a carbon‐hydrogen bond the associated hydrogen will be lost from the surface.

Above 900 K, for all four molecules, the intensity of H_2_ in the mass spectrometer is back at the baseline level, see Figure 8, indicating no more H_2_ is desorbing from the Cu(111) surface. At 900 K, we therefore expect the molecules to have lost all hydrogen atoms and have formed a possibly, disordered graphene‐like network on the surface.^[^ ^93^, ^94^, ^95^, ^96^, ^97^, ^98^ ^]^ This is consistent with the general behavior of organic molecules on metal surfaces at high temperatures.^[^ ^99^, ^100^, ^101^ ^]^

**Figure 7:**
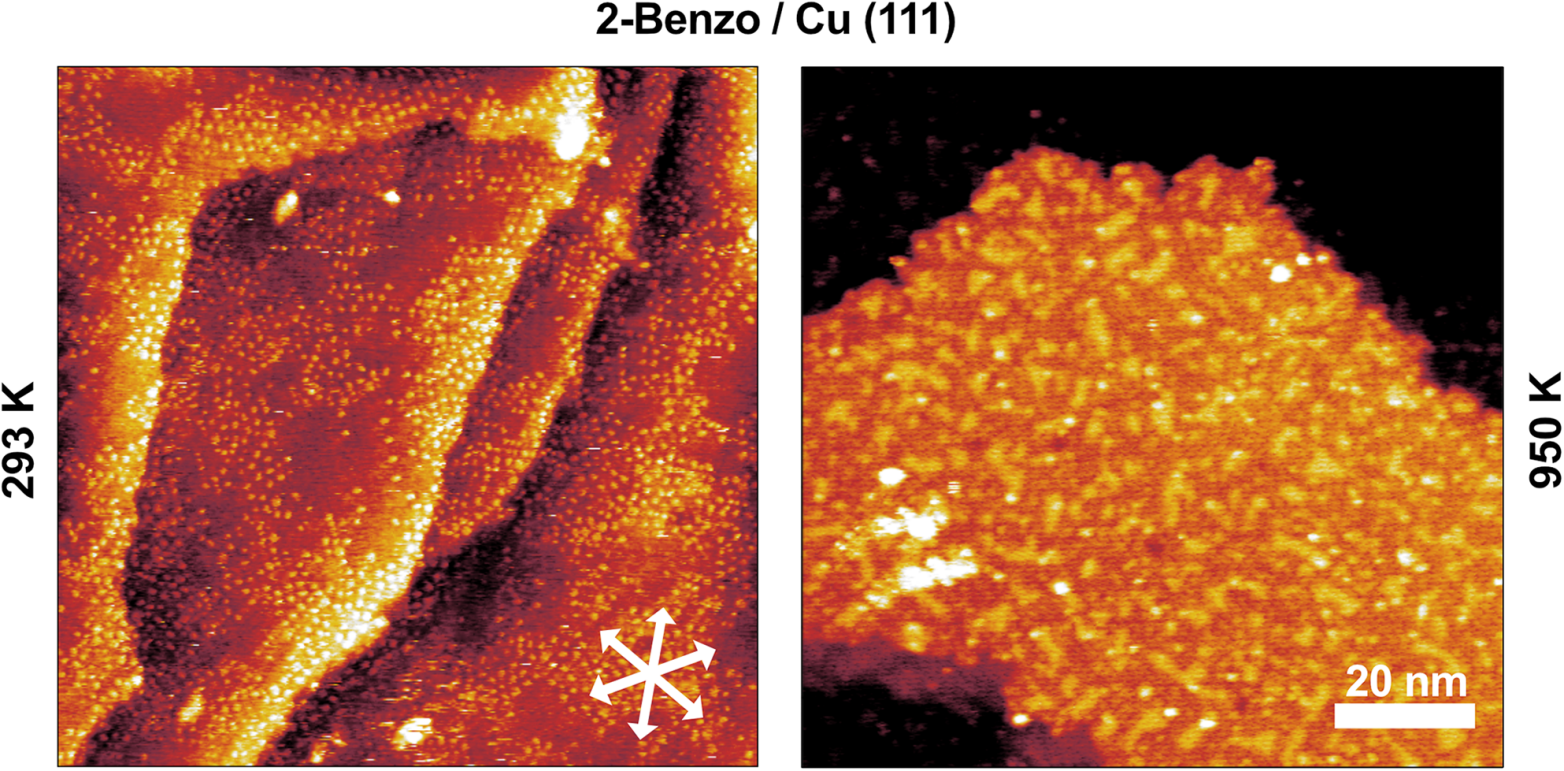
STM images of slightly more than one closed layer of 2‐Benzo, as deposited at room temperature (left), and after 1 minute at 950 K (right). Scale bar and substrate direction apply to both STM measurements. Before annealing, single molecules in the 2nd layers appear as individual bright protrusions. After annealing to 950 K, the polymerization reaction, visible in TPD between 650 and 900 K, see Figure 2, has left a highly disordered structure of irregular shapes on the surface. A polynomial background was subtracted for the room temperature image to better show the adsorption behavior across all the terraces. (293 K: −0.92 V, 50 pA, 950 K: −1.44 V, 37 pA).

Figure 7 shows STM images of slightly more than one closed layer of 2‐Benzo molecules, as deposited at room temperature (left), and after annealing for 1 minute at 950 K (right). An interesting minor detail is that, against our expectations, at room temperature, the second‐layer 2‐Benzo molecules appear immobile. This could potentially be a consequence of the upright‐standing isoindole groups of the free‐base molecules in the first layer, see Figure 3 top left, interacting with the molecules in the second layer, anchoring them in place.

After heating the slightly more than one closed layer of 2‐Benzo molecules to 950 K, we observe an irregular structure on the surface, where individual molecules can no longer be identified. We believe this to be a fully‐polymerized highly‐disordered graphene‐like network on the surface.

### 3.4 Reaction Rates

For 2‐Benzo, polymerization, leading to complete dehydrogenation, would result in the desorption of 12 H_2_ molecules per porphyrin molecule, giving an expected H_2_ desorption ratio of 1 : 4 : 12 between the three reactions. Shading the areas corresponding to those ratios, gives the magenta, orange, and blue areas in Figure 2, and a very good match to the three main features of the TPD spectrum.

A direct comparison of the STM and TPD temperatures is difficult both because the sample holders and thermocouple mountings are different for the two UHV setups, but also because the TPD spectra are obtained with a heating rate of 5 K/s whereas the STM measurements are measured after up to 5 minutes annealing at the denoted temperatures. Nevertheless, if we look at the STM images in Figure 2, we can tell, based on the lower density of immobile free‐base molecules at 373 K (top right) compared to 293 K (top left), that we go from roughly 50% metalation at 373 K, to an almost complete breakup of the islands at 423 K (bottom right), which is a temperature range of only 50 K.

If we look at the TPD spectrum in Figure 2, and start at the peak of the metalation at 410 K (magenta region), corresponding to roughly 50% metalation, 50 K will take us only into the first roughly 20% of the broad ring‐fusion peak (orange region). This means an average of one ring fusion per molecule is enough to destabilize the islands.

Assuming four ring fusions per molecule, to avoid forming bowl‐shaped molecules, we can calculate the expected H_2_ desorption ratios for metalation, ring fusion, and polymerization to be 1 : 4 : 10 for 0‐Benzo, 1 : 4 : 12 for 2‐Benzo, 0 : 4 : 12 for Cu 2‐Benzo, and 1 : 4 : 14 for 4‐Benzo. If we shade those areas on the H_2_ desorption curves of the completed first layer of all four molecules, see Figure 4, we get a very reasonable agreement between desorption minima and the expected reactions.

**Figure 8:**
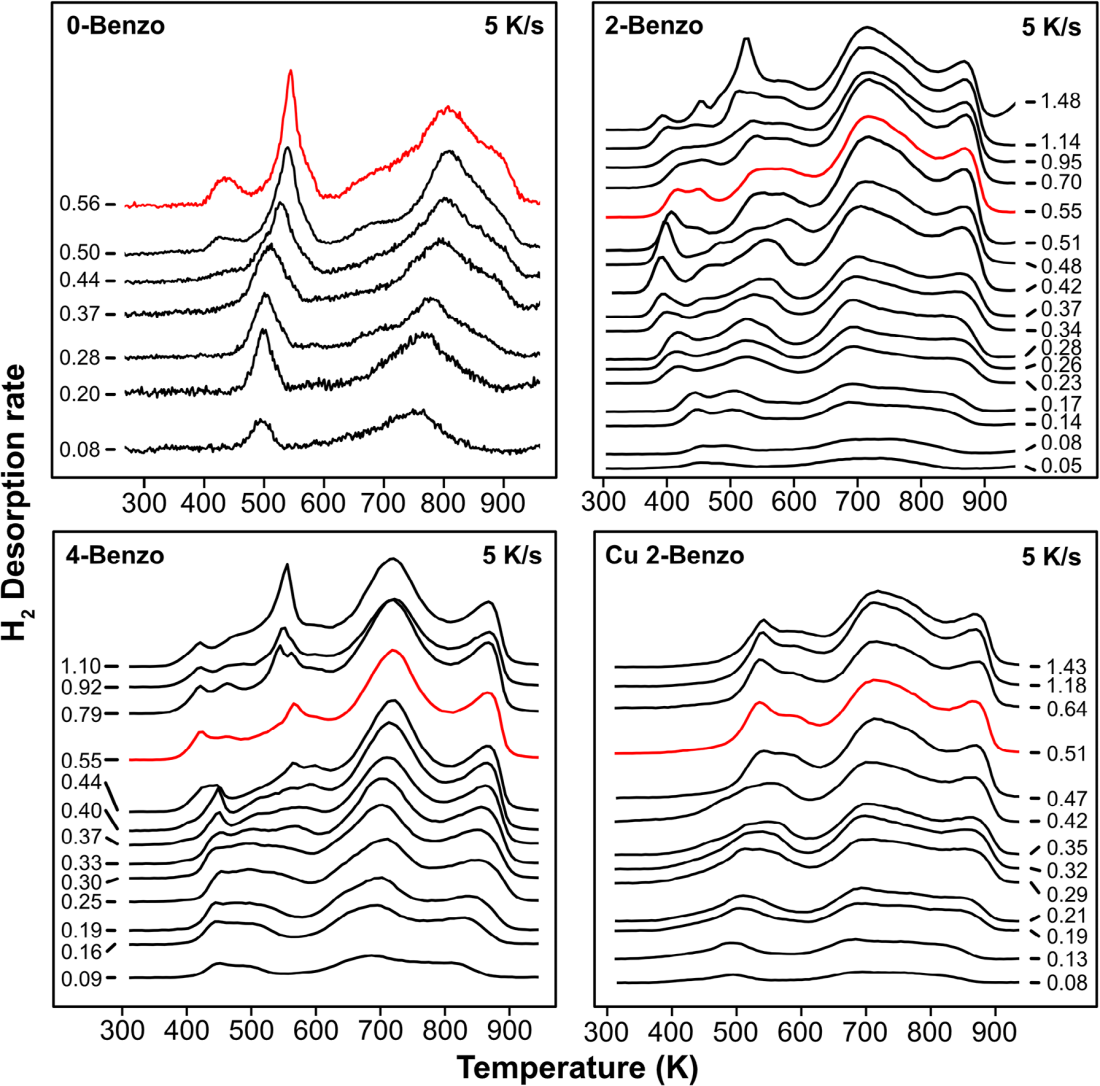
Selected TPD spectra of the four different benzoporphyrins. The numbers next to the traces indicate coverages in molecules/nm^2^, and the red traces indicate the coverage closest to the fully covered surface. All traces below the fully covered traces in red are plotted with a y‐axis offset proportional to their coverages, higher coverages are offset for a visually pleasing appearance. The complete TPD series with marked integration points can be found in the Supporting Information Figure S1. The spectra of 0‐Benzo were previously published by Röckert et al.^[^ ^63^ ^]^

This strongly suggests that the three reactions are mostly sequential: The molecules first metalate, then they ring fuse, and finally they polymerize. This is supported by the N 1 s XPS spectra in Figure 4, measured at 500 K, all showing a single N 1 s feature, indicative of a metalated molecule. It is further supported by the TPD spectra of 2H 2‐Benzo and Cu 2‐Benzo in Figure 5 and, for the full coverage range, Figure 8.

Figure 8 shows the H_2_ desorption spectra of the studied molecules in four waterfall plots, where the red curves indicate the coverage closest to the fully covered surface: 0.56 molecules/nm^2^ for 0‐Benzo, 0.53 molecules/nm^2^ for 2H, and Cu 2‐Benzo and 0.54 molecules/nm^2^ for 4‐Benzo, these are the spectra also shown in Figure 4. For coverages less than the red curves, the vertical offset is proportional to the coverage. For the few curves with higher coverages, a uniform and more aesthetically‐pleasing spacing is used instead.

For 0‐Benzo and 4‐Benzo there is an abrupt change in the onset temperature of H_2_ desorption at coverages just below the red spectra, corresponding to the completed first layers, see Figure 8. Because the initial desorption of H_2_ originates from the metalation reaction, this means that the initial rate of metalation increases abruptly for 0‐Benzo and 4‐Benzo as the coverage is increased from 0.30 to 0.55 molecules/nm^2^.

For ring fusion and polymerization, there is a slow and steady shift of the observed features to higher temperatures with increasing coverage for all four molecules.

However, the desorption curves in Figure 8 are very complex with multiple overlapping features. To create a better overview, we are therefore going to use the sequential behavior of the reactions to estimate the temperatures corresponding to 50% metalation, 50% ring fusion, and 50% polymerization. We did this by integrating the desorption curves. As an example, for 2‐Benzo the integrated areas of metalation, ring fusion, and polymerization should have the ratio 1 : 4 : 12. The temperature corresponding to 1 / 34^th^ of the total area, therefore, corresponds to 50% metalation, 3 / 17^th^ corresponds to 50% ring fusion, and 11 / 17^th^ to 50% polymerization.

**Figure 9:**
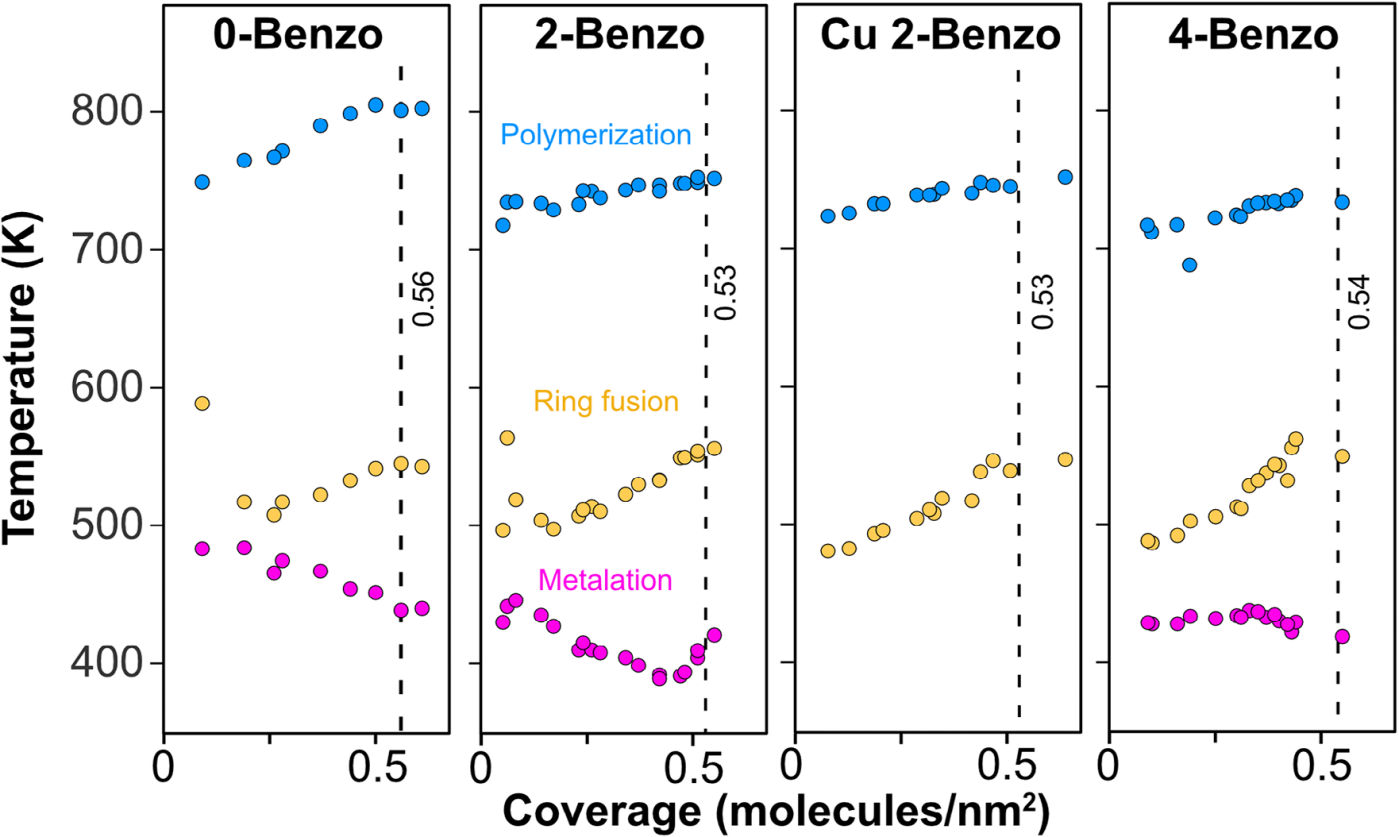
Coverage‐dependent reaction temperatures of metalation, ring fusion, and polymerization up to coverages slightly above the closed layer. The coverages of the closed layers are marked by the dashed lines. The colored dots indicate the temperatures, where, according to the integration of the TPD spectra, the three reactions (metalation, ring fusion, and polymerization) are 50% complete. The three free‐base porphyrins show completely different behaviors in their coverage‐dependent metalation behavior, while the trends in the ring fusion and polymerization reactions of all investigated tetraphenylporphyrins are rather similar.

This yields the coverage‐dependent reaction temperatures of the metalation, ring fusion, and polymerization reactions shown in Figure 9. To see the integration points marked in the desorption curves we refer the reader to Figure S1 in the Supporting Information. While, as already mentioned, ring fusion, and polymerization behave quite similar for all four molecules, there are significant differences in the coverage behavior of the metalation reaction:

For 4‐Benzo, see Figure 9, the temperature for 50% metalation (magenta) stays constant at ∼435 K until it decreases slightly by 15 K to ∼420 K, just before the completed first layer, which is indicated as a dashed vertical line. Very‐few STM images exist in the literature of this molecule on Cu(111),^[^ ^102^ ^]^ but from those, we know of two densely‐packed adsorption structures. The small decrease in the 50% metalation temperature, we observe just below the completed first layer, could be related to a transition from one adsorption structure to another.

For 0‐Benzo, see Figure 9, the temperature for 50% metalation decreases linearly with coverage form ∼485 K by 40 K, until it levels off at ∼445 K at the completed first layer and stays constant into the multilayers. This decrease has previously been associated with a change in adsorption structure from individual isolated molecules to a checkerboard adsorption structure, which starts at 0.30 molecules/nm^2^ and eventually covers the whole surface.^[^ ^22^, ^23^, ^25^ ^]^

For 2‐Benzo, see Figure 9, the temperature for 50% metalation initially decreases linearly with coverage from ∼445 to ∼390 K, until it abruptly increases again to ∼425 K, just before the completion of the first layer. The initial linear decrease is very similar to that of 0‐Benzo, but metalation occurs at overall lower temperatures for 2‐Benzo. Also similar to 0‐Benzo, we observe the initial formation of ordered islands at ∼0.30 molecules/nm^2^, but the islands are difficult to resolve.^[^ ^30^ ^]^

The integration analysis method is a strong simplification of a very complex series of behaviors, but when comparing the integration points with the features of the desorption curves, see Figure S1, there is a very clear correlation between the two. As discussed above, we can also link the abrupt changes in the integration temperatures to observed changes in the adsorption structures.

**Figure 10:**
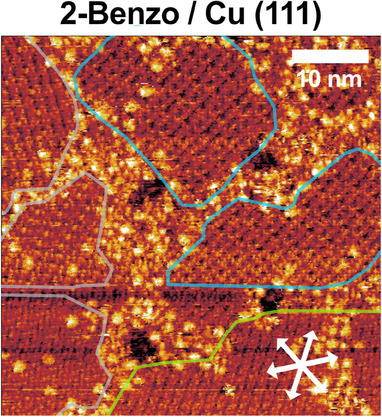
STM image of 0.55 molecules/nm^2^ 2‐Benzo on Cu(111) directly after deposition at room temperature. Different domains are visible, some where the molecular rows follow the substrate direction (light green) and some, with a different appearance, that are rotated by 30° (blue). Additional smaller, hard‐to‐assign domains (grey) can also be seen. (−1.51 V, 73 pA).

Close to the completed first layer, we observe a coexistence of multiple adsorption structures of 2‐Benzo in STM, which fall into two different categories: (i) rows along the atomic rows of the Cu(111) substrate and (ii) rows aligned by 30° relative to the atomic rows of the substrate. Figure 10 shows the best‐resolved STM image we have of these coexisting structures. The domains encircled in green have rows in the direction of the substrate rows (type i), with no submolecular details within the rows. This could simply be a compressed structure of molecules in the same conformation as the individual isolated molecules, which at lower coverages are aligned along the atomic rows of the substrate and exhibit no submolecular details in STM, see Shaker et al.^[^ ^30^ ^]^ (Figure 9). Because the molecule, in this conformation, is completely planar, except for the upright‐standing isoindole groups, the molecule–molecule interactions should be repulsive and the structure should get less and less stable with increasing coverage.

The domains encircled in blue are aligned by 30° relative to the atomic rows of the substrate (type ii) and display some submolecular details. If this is the higher‐coverage structure, it would make sense for the molecules to have a conformation that would allow for attractive T‐type interactions between the molecules. One possibility is the structure adopted by the metalated molecules, with attractive T‐type interactions between phenyl rings and isoindole groups of adjacent molecules,^[^ ^30^ ^]^ but that structure should appear as sharp double protrusions in the STM image, which is not what we observe for the blue structure. However, during the DFT search for the energetically most‐stable isolated adsorption structure of 2‐Benzo on Cu(111), we came across several saddle‐shape conformations, the most stable being only 0.33 eV less stable than the most‐stable inverted structure. For details about the saddle‐shape structures we refer to our previous study (Shaker et al.).^[^ ^30^ ^]^

A saddle‐shape deformation should allow for attractive T‐type interactions between molecules, and in the simulated STM images the saddle‐shaped molecules all appear dark in the center with four brighter protrusions, corresponding to the four phenyl rings. The dark features in the blue domains, see Figure 10, could therefore be the dark centers of saddle‐shaped molecules.

## 4 Conclusion

We have used STM, X‐ray photoelectron spectroscopy (XPS), TPD, and DFT calculations to follow the coverage‐dependent thermal reactions (metalation, ring fusion, and polymerization) from 280 to 1000 K of three benzoporphyrins: tetraphenyltrandisbenzoporphyrin (2‐Benzo), Cu 2‐Benzo, and tetraphenyltetrabenzoporphyrin (4‐Benzo) with Cu(111), and we compared this with previous data^[^ ^22^, ^63^ ^]^ of tetraphenylporphyrin (0‐Benzo) on Cu(111).

Using STM, we have demonstrated how the molecular arrangement of the free‐base dibenzo molecules on the surface changes with increasing temperature, from isolated, rather‐immobile molecules at room temperature to elongated ordered islands with a few mobile molecules at 373 K. As the temperature is increased further, we have seen how the ordered islands become rough and irregular at 398 K, before completely breaking up into mobile molecules at 423 K, eventually creating a very rough and disordered structure at 950 K.

With XPS, we can confirm that the first reaction step, creating the ordered islands, is metalation, clearly visible in the N 1 s region. This conclusion is unambiguously confirmed when comparing the H_2_ desorption traces of the free‐base and metalated dibenzo molecules.

With TPD, we have followed the rates of the different surface reactions via the rates of H_2_ desorption from the surface, giving us data with very‐good coverage and temperature resolution. For all three free‐base molecules, the rate of metalation is highest either at or just below the completed first layer, often with fairly abrupt changes of the rate as a function of coverage, the latter are associated with changes in the adsorption structures of the molecules on the surface.

The roughening and eventual breaking up of the metalated dibenzo‐islands, observed in STM, is likely associated with partially ring‐fused molecules attaching to the ends of the molecular rows, making it easier to terminate the rows and thereby creating rougher islands. As the degree of ring fusion increases and all the molecules are at least partially ring‐fused, there are no interactions left to hold the islands together, and they break up into mobile molecules, creating stripy features in the STM images.

As the temperature is increased further, the mobile molecules begin to polymerize, eventually creating a very‐rough structure on the surface at 950 K.

For all four molecules, the rates of ring fusion and polymerization gradually decrease with increasing coverage.

## Conflict of Interest

The authors declare that they have no known competing financial interests or personal relationships that could have appeared to influence the work reported in this paper.

## Supporting information



Supporting Information

## Data Availability

All data used in this study are available from the Zenodo open repository at https://doi.org/10.5281/zenodo.15007016, reference number 15 007 016.
