# Supplementary material for: Coverage‐ and Temperature‐Dependent Rates of Metalation, Ring Fusion, and Polymerization of Benzoporphyrins on Cu(111)
Source: Chemistry. 2025 Jul 1;31(40):e202500998. doi: 10.1002/chem.202500998 (PMC12272011; doi:10.1002/chem.202500998)
Supplement: Supplementary file 1 — Supporting Information [file CHEM-31-e202500998-s001.pdf]

Supporting Information

for

Coverage- and Temperature-Dependent Rates of Metalation, Ring Fusion and Polymerization of Benzoporphyrins on Cu(111)

Maximilian Muth,[a] Majid Shaker,[a] Julien Steffen,[b] Alexander Wolfram,[a] Simon Steinbach,[a] Lampros-Pascal Gazetas,[c] Norbert Jux,[c] Andreas Görling,[b,d] Hans-Peter Steinrück,[a] Ole Lytken[a,*]

[a] Lehrstuhl für Physikalische Chemie II, Friedrich-Alexander-Universität Erlangen-Nürnberg, Egerlandstr. 3, Erlangen 91058, Germany

[b] Lehrstuhl für Theoretische Chemie, Friedrich-Alexander-Universität Erlangen-Nürnberg, Egerlandstr. 3, Erlangen 91058, Germany

[c] Lehrstuhl für Organische Chemie II, Friedrich-Alexander-Universität Erlangen-Nürnberg, Nikolaus-Fiebiger-Str. 10, Erlangen 91058, Germany

[d] Erlangen National High Performance Computing Center (NHR@FAU), Martensstr. 1, D-91058 Erlangen, Germany

Figure S1 gives an overview of all the recorded TPD spectra used for the evaluation in the main article.

The colored dots indicate the temperatures, where according to integration of the TPD spectra (bottom) the reactions (metalation, ring fusion and polymerization) should be 50 % completed. The colored triangles indicate where 100 % of the reaction should have taken place.

The spectra in red marks a full monolayer, and all spectra below this coverage were plotted with y-axis offsets proportional to their coverages (in molecules/nm^2). All spectra above this coverage were spaced more evenly, independent of coverages.

The TPD spectra of 0-Benzo in Figure S1 was previously published by Röckert et al.[1] For these spectra the areas of the TPD spectra were normalized to the coverage on the surface, as determined by the C 1s : Cu $2p_{3/2}$ signal ratio. This was done to correct for an irreproducible distance between the sample and the Feulner cup, that our early TPD measurements suffered from. A few of our 2-Benzo spectra, suffered from the same problem and had to be corrected the same way.

Some of the 0-Benzo and 2-Benzo TPD spectra show an exponential-like increase in H_2 desorption starting at about 950 K. This is most likely caused by an inefficient liquid-nitrogen cooling of the manipulator, causing the sample holder to warms up, leading to additional hydrogen desorption.

Figure S2 shows mass spectra of 2-Benzo and Cu 2-Benzo being directly evaporated into a Balzers QMA 400 quadrupole mass spectrometer.

The red spectra were recorded right after loading the evaporator with fresh porphyrins without any previous degassing. The blue spectra were recorded after ~5 min degassing. The black spectra were recorded just before the evaporator has become empty. The molecular signal of 2-Benzo appears at m/z=714. The signal at m/z=664 has a difference of m/z=50 to the main signal, which fits perfectly with one missing benzo group. This suggests that there is an impurity of benzoporphyrin molecule with only one benzo group (1-Benzo) present. In the mass spectr*a*, the proportion of this 1-Benzo impurity is given in percent. Additional*l*y, the spectra show signals at m/z=635.5 and 559. These signals are most probably related to cleaved-off phenyl rings.

The molecular signal of Cu 2-Benzo is visible at m/z=775. Also, Cu 2-Benzo shows impurities of molecule with only one benzo group (Cu 1-Benzo m/z=725). Here again, we assign the signals at m/z=696 and 619 to missing phenyl rings.

The 1-Benzo / Cu 1-Benzo impurities stems from not-fully-reacted early precursor compounds which were apparently not fully separated from the desired molecule. This is likely due to similar polarities of the porphyrin compounds.

Due to longer degassing times used before the performance of the experiments in the main article, we expect these impurities to lie between the blue and the black mass spectra. For 2-Benzo the 1-Benzo impurity is therefor expected to lie between 13 and 11%. For Cu 2-Benzo the Cu 1-Benzo impurity we estimate the impurity to in a range between 17 and 4 %.

Figure S3 shows a top and side view of the T-type interactions within the DFT-calculated Cu 2-Benzo structure from Figure 3.

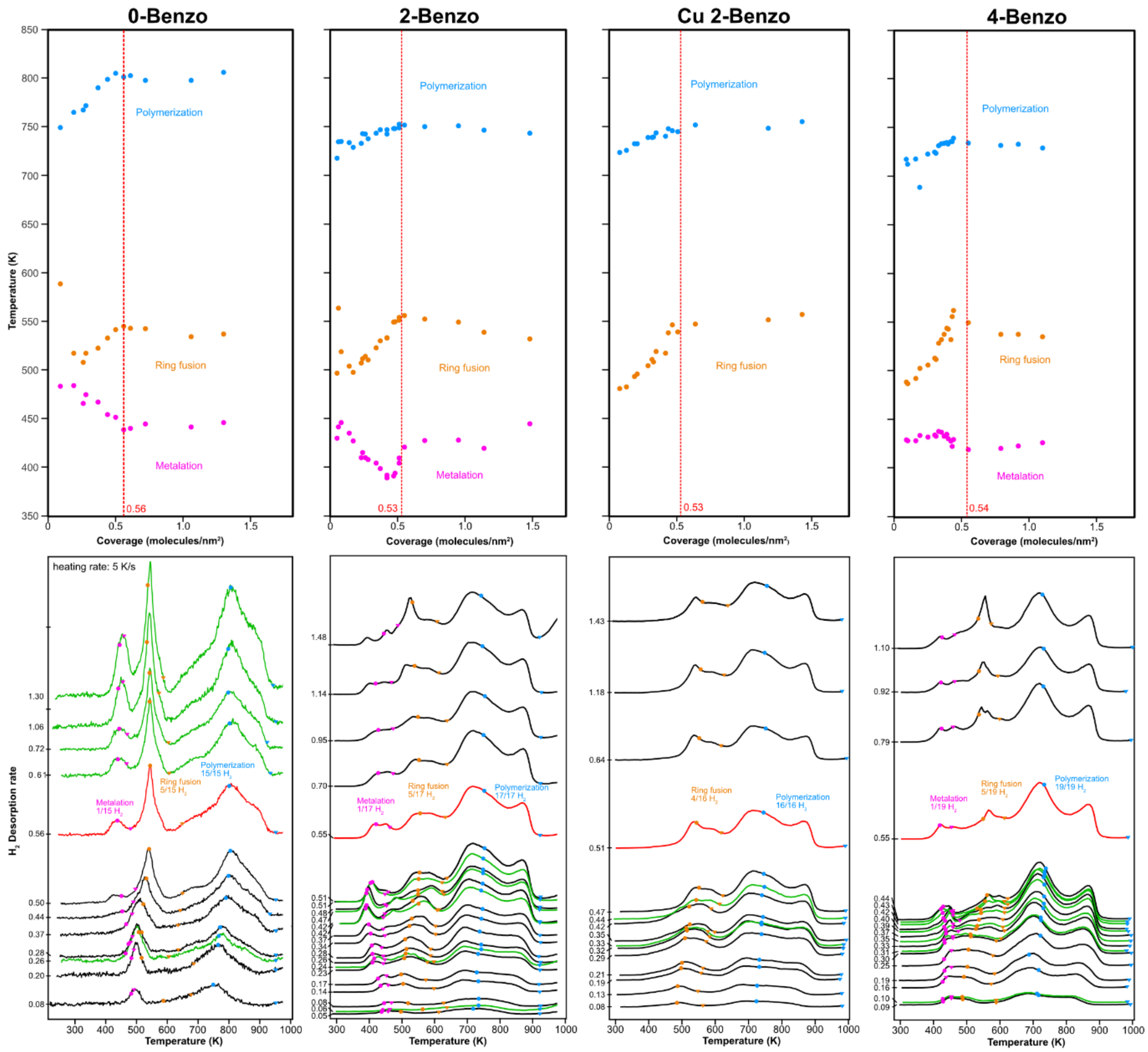

Figure S1: Overview of all the recorded spectra used to show coverage dependence of the reaction temperatures of metalation, ring fusion and polymerization. The colored dots indicating the temperatures where according to integration of the TPD spectra 50% (dots) and 100% (triangles) have taken place. The green spectra were removed from Figure 8 in the main article for a clearer overview.

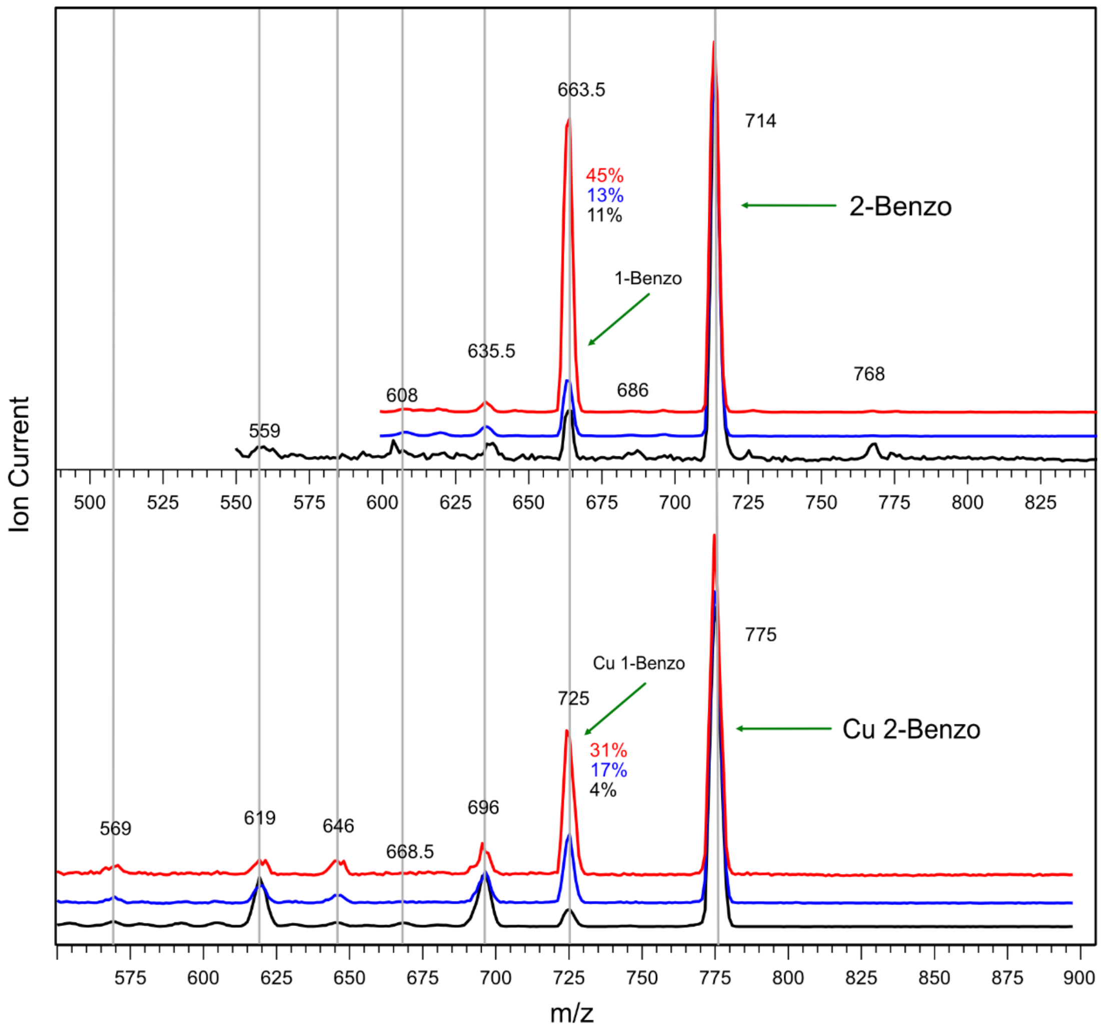

Figure S2: Mass spectra of 2-Benzo and Cu 2-Benzo. For Cu 2-Benzo a wider scanning range was used. The red spectra were recorded right after loading the evaporator with fresh porphyrins without any previous degassing. The blue spectra were recorded after ~5 min degassing. The black spectra were recorded just before the evaporator has become empty. Due to longer degassing times used before the performance of the experiments performed in the main article, we expect impurities in between the blue and the black mass spectra.

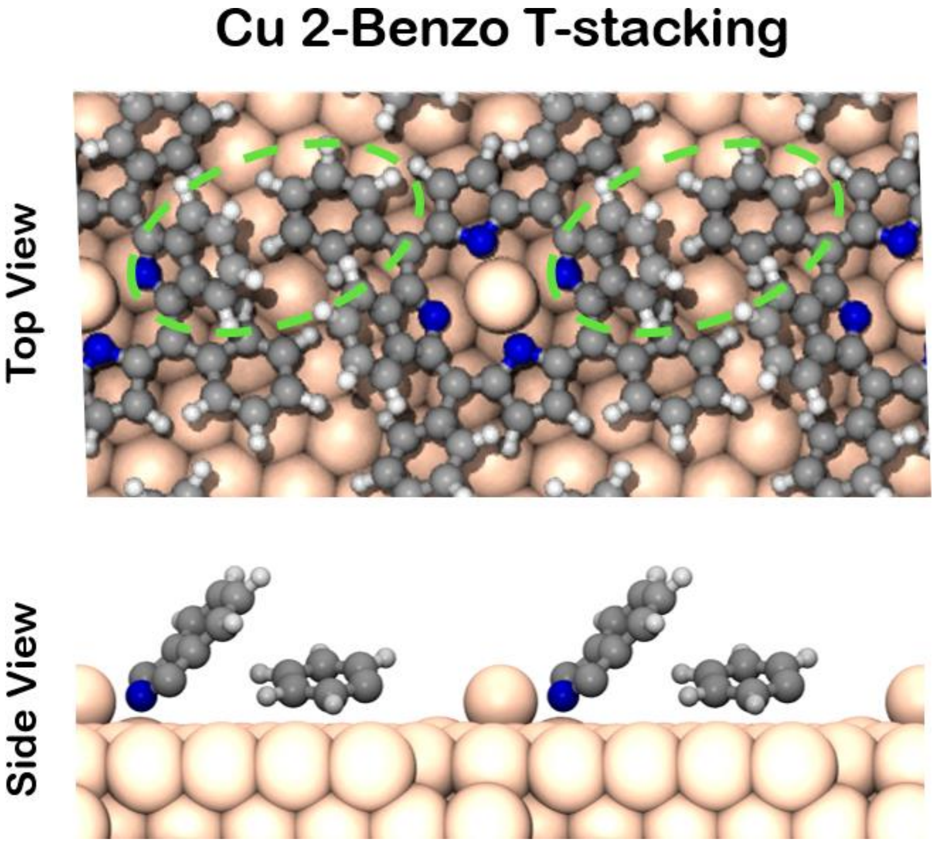

Figure S3: Top and side views of the T-type interactions between the isoindole and phenyl rings in the DFT-calculated adsorption structure of Cu 2-Benzo on Cu(111). For ease of viewing, only the adjacent isoindole and phenyl rings marked by the dashed circles in the top view are shown in the side view. From the top and side views, it is clear that the isoindole groups are tilted upwards, while the phenyl rings are flatter lying, and that the isoindole groups and phenyl rings are in close proximity and facing each other, indicative of a T-type interaction.

References

1. Röckert, M.; Franke, M.; Tariq, Q.; Ditze, S.; Stark, M.; Uffinger, P.; Wechsler, D.; Singh, U.; Xiao, J.; Marbach, H.; Steinrück, H.-P.; Lytken, O., Coverage- and Temperature-Dependent Metalation and Dehydrogenation of Tetraphenylporphyrin on Cu(111). *Chem. Eur. J.* **2014,** *20*, 8948 – 8953.
